# Finding a most parsimonious or likely tree in a network with respect to an alignment

**DOI:** 10.1007/s00285-018-1282-2

**Published:** 2018-08-19

**Authors:** Steven Kelk, Fabio Pardi, Celine Scornavacca, Leo van Iersel

**Affiliations:** 1 Department of Data Science and Knowledge Engineering (DKE), Maastricht University, P.O. Box 616, 6200 MD Maastricht, The Netherlands; 2 LIRMM, Université de Montpellier, CNRS, Montpellier, France; 3 Institut des Sciences de l’Evolution, CNRS, IRD, EPHE, Institut de Biologie Computationnelle (IBC), Université de Montpellier, 34095 Montpellier Cedex 5, France; 4 Delft Institute of Applied Mathematics, Delft University of Technology, Van Mourik Broekmanweg 6, 2628 XE Delft, The Netherlands

**Keywords:** Phylogenetic tree, Phylogenetic network, Maximum parsimony, Maximum likelihood, NP-hardness, APX-hardness, 92D15, 68Q25, 92D20

## Abstract

Phylogenetic networks are often constructed by merging multiple conflicting phylogenetic signals into a directed acyclic graph. It is interesting to explore whether a network constructed in this way induces biologically-relevant phylogenetic signals that were not present in the input. Here we show that, given a multiple alignment *A* for a set of taxa *X* and a rooted phylogenetic network *N* whose leaves are labelled by *X*, it is NP-hard to locate a most parsimonious phylogenetic tree displayed by *N* (with respect to *A*) even when the level of *N*—the maximum number of reticulation nodes within a biconnected component—is 1 and *A* contains only 2 distinct states. (If, additionally, gaps are allowed the problem becomes APX-hard.) We also show that under the same conditions, and assuming a simple binary symmetric model of character evolution, finding a most likely tree displayed by the network is NP-hard. These negative results contrast with earlier work on parsimony in which it is shown that if *A* consists of a single column the problem is fixed parameter tractable in the level. We conclude with a discussion of why, despite the NP-hardness, both the parsimony and likelihood problem can likely be well-solved in practice.

## Introduction

Rooted phylogenetic networks are generalizations of rooted phylogenetic trees that allow horizontal evolutionary events such as horizontal gene transfer, recombination and hybridization to be modelled (Huson et al. 2010; Morrison 2011; Gusfield 2014). This is achieved by allowing nodes with indegree 2 or higher, known as *reticulation* nodes. Recent years have seen an explosion of interest in constructing rooted phylogenetic networks, fuelled by the growing awareness that *incongruence* in phylogenetic and phylogenomic datasets is not simply a question of evolutionary “noise”, but sometimes the result of evolutionary phenomena more complex than speciation and mutation (e.g. Zhaxybayeva and Doolittle 2011; Abbott et al. 2013; Vuilleumier and Bonhoeffer 2015).

Although many modelling questions surrounding the construction of phylogenetic networks are still to be answered, it is commonplace to associate a rooted phylogenetic network with the set of rooted phylogenetic trees that it contains (“displays”). Informally speaking, a rooted phylogenetic network displays a rooted phylogenetic tree if the tree can be topologically embedded inside the network. A network is not necessarily defined by the set of trees it displays, but the notion of display is nevertheless a recurring theme in the literature, since networks themselves are often constructed by merging phylogenetic trees subject to some optimality criterion. It is well-known that it is NP-hard to determine whether a network displays a given tree (Kanj et al. 2008), although on many restricted classes of phylogenetic networks the problem is polynomial-time solvable (Van Iersel et al. 2010; Fakcharoenphol et al. 2015; Gambette et al. 2015).

Although it is important to be able to determine whether a network displays a given tree, we may also wish to ask what the “best” tree is within the network, subject to some optimality criterion. For example, given a network *N* and a multiple alignment *A*, we may wish to ask for a tree *T* displayed by *N* with lowest parsimony score with respect to *A*. Similarly, if the network is decorated by edge lengths or probabilities, we may wish to identify a most *likely* tree displayed by the network, that is, a tree that maximizes the probability of generating *A*, under a given model of evolution. Such questions are natural, as the two following examples show. First, phylogenetic networks are often constructed by topologically merging incongruent phylogenetic signals (e.g. Kelk and Scornavacca 2014), and it is insightful to ask whether the network thus constructed displays trees which have interesting properties (such as a low parsimony score or a high likelihood) which were not in the input. Second, we may wish to perform classical phylogenetic tree construction under criteria such as maximum parsimony or maximum likelihood (e.g. Jin et al. 2006, 2007), but within the restricted space of trees displayed by a given network.

Problems of the above kind are already known to be NP-hard, since it is NP-hard to determine a most parsimonious tree *T* displayed by a given network *N* even when the alignment *A* consists of a *single* column and the network is binary (Fischer et al. 2015). However, the gadgets used in that hardness reduction produce networks with very high *level*, where level is the maximum number of reticulation nodes in a biconnected component of the network. On the positive side, the same article shows that the problem on an alignment consisting of a single column is FPT in the level of the network. This means that, on a network with level *k*, the problem can be solved in time $f(k) \cdot \text {poly}(n)$ where *f* is a function that depends only on *k* and *n* is the size of the network. Such results are useful in practice, when (as is often the case) *k* is small.

The question emerges whether the positive FPT result goes through when *A* does not consist of a single column, but potentially many columns – a problem introduced more than one decade ago (Nakhleh et al. 2005). Here we show that this is not the case. We prove the rather negative result that locating a most parsimonious tree in a rooted binary network *N* is NP-hard, even under the following restricted circumstances: (1) each biconnected component of the network contains exactly one reticulation node (i.e. is “level-1”); (2) each biconnected component of the network has exactly three outgoing arcs; (3) the alignment *A* consists of two states. If indel symbols are permitted then the problem is not only NP-hard, but also difficult to approximate well (APX-hard). If any of the conditions (1)-(3) are further strengthened (respectively: the network becomes a tree; the reticulation nodes become redundant; the alignment becomes uninformative), the problem becomes trivially solvable, so in some sense this is a “best possible” (or the “worst possible”, depending on your perspective) hardness result. Next, we consider the question of identifying a most likely tree in the network. We obtain NP-hardness under the same restrictions (1)-(3), subject to the simple binary symmetric model of character evolution. It is no coincidence that restrictions (1)-(3) again apply, since the hardness of the likelihood question is established by reducing the parsimony variant of the problem to it. Specifically, we show that a most likely tree displayed by a network with sufficiently short branches is necessarily also a most parsimonious tree.

Although the main results in this paper are negative, some reasons for hope are given in the conclusion.

## Preliminaries

A *rooted binary phylogenetic networkN* on a set *X* of taxa is a directed acyclic graph where the leaves (nodes of indegree-1 and outdegree-0) are bijectively labelled by *X*, there is a unique root (a node of indegree-0 and outdegree-2) and all other nodes are either tree nodes (indegree-1 and outdegree-2) or reticulation nodes (indegree-2 and outdegree-1). For brevity we henceforth simply use the term *network*. A *rooted binary phylogenetic tree* (henceforth *tree*) is a phylogenetic network without any reticulation nodes. A *cherry* is a pair of taxa that share a common parent. A *rooted binary caterpillar* is a tree with exactly one cherry.

The *level* of a network *N* is the maximum number of reticulation nodes in a biconnected component of the undirected graph underpinning *N*. In this article we will focus exclusively on level-1 networks. In level-1 networks, maximal biconnected components that are not single edges are simple cycles that contain exactly one reticulation node; such biconnected components are called *galls*. An arc whose tail (but not head) is a node of a gall is called an *outgoing* arc.

A *characterf* is a surjective mapping $f: X \rightarrow S$ where *S* is a set of discrete *states*. When *S* contains two states we say that *f* is a *binary* character. Given a tree $T = (V,E)$ and a character *f*, both on *X*, we say that $\hat{f}: V \rightarrow S$ is an *extension* of *f* to *T* if $\hat{f}(x) = f(x)$ for all $x \in X$. The *number of mutations* induced by $\hat{f}$ (on *T*), denoted $l_{\hat{f}}(T)$, is the number of edges $\{u,v\} \in E$ such that $\hat{f}(u) \ne \hat{f}(v)$. The *parsimony score* of *f* with respect to *T*, denoted $l_f(T)$, is the minimum number of mutations induced ranging over all extensions $\hat{f}$ of *f*. Any extension that achieves this minimum is called an *optimal* extension. An optimal extension can be computed in polynomial time using Fitch’s algorithm (Fitch 1971), which for completeness we describe in the appendix along with some of its relevant mathematical properties. (Note that there potentially exist optimal extensions that cannot be generated by Fitch’s algorithm.)

For a network *N* and a tree *T*, both on *X*, we say that *NdisplaysT* if there exists a subtree $T'$ of *N* such that $T'$ is a subdivision of *T*. An equivalent definition of “displays” relies on the notion of a *switching*, where a switching is a subtree $N'$ of *N* obtained by, for each reticulation node *u*, deleting exactly one of *u*’s incoming edges. *N* displays *T* if and only if there exists some switching $N'$ of *N* and a subdivision $T'$ of *T* such that $T'$ is a subgraph of $N'$. In both definitions we say that $T'$ is an *image* of *T* inside *N*.

The softwired parsimony score^1^ of a network *N* with respect to *f* is the minimum, ranging over all trees *T* displayed by *N*, of $l_f(T)$.

We now extend the above concepts to *alignments*. An alignment *A* is simply a linear ordering of characters. In this paper the linear ordering is irrelevant so we can arbitrarily impose an ordering and write $f \in A$ without ambiguity. An alignment can naturally be represented as a matrix with |*X*| rows and |*A*| columns; we therefore use the terms characters and columns interchangeably (and, following the use of alignments in practice, we sometimes refer to the rows of the matrix as *sequences*). The parsimony score of a tree *T* with respect to *A*, denoted $l_{A}(T)$, is simply $\sum _{f \in A}l_f(T)$.

When extending this concept to networks, two definitions have been proposed: the parsimony score of a network with respect to an alignment *A*, denoted $l_{A}(N)$, can be defined as

1. $$\displaystyle \sum _{f \in A} \displaystyle \min _{T \in \mathcal {T}(N)}l_f(T)$$
  or
2. $$\displaystyle \min _{T \in \mathcal {T}(N)}\sum _{f \in A}l_f(T)$$

where $\mathcal {T}(N)$ is the set of trees displayed by the network. According to the first definition (introduced in Hein (1990)), each character can follow a different tree displayed by the network, while in the second one (introduced in Nakhleh et al. (2005)) all characters of the alignment follow the same tree. In this paper, we will adopt the latter definition, and a tree *T* that is the minimizer of this sum is called a *most parsimonious (MP) tree displayed by N* (with respect to *A*).

Note that in applied phylogenetics alignments often contain indels, encoded using a gap symbol “-”. From the parsimony perspective it is not uncommon to treat these symbols as wildcards that do not induce mutations; the taxon “does not care” what state it is assigned. (Note however that extensions are *not* allowed to contain gap symbols). To compute $$l_f(T)$$ when a character $$f: X \rightarrow S$$ maps some of its taxa to the gap symbol, we can run Fitch’s algorithm with a slight modification to the bottom-up phase: for each taxon *x* such that $$f(x) = $$“-”, we assign the entire set of states *S* to *x*. Moreover, as the following observation shows, the use of “-” symbols does not make the problem of identifying a most parsimonious tree displayed by a network significantly harder.

### Observation 1

Let *A* be an alignment for a set of taxa *X* and let *N* be a phylogenetic network on *X*. Suppose *A* uses the states $\{0,1,\text {``-''}\}$. Let *k* denote the total number of gap symbols in *A*. In polynomial time we can construct an alignment $A'$ on 2|*X*| taxa, which uses only states $\{0,1\}$, and a network $N'$ on 2|*X*| taxa, such that there is a polynomial-time computable bjiection *g* mapping trees displayed by *N* to trees displayed by $N'$. This bijection *g* has the property that, for each tree *T* displayed by *N*, $l_{A'}( g(T) )= l_A(T) + k$. Consequently, *T* is a most parsimonious tree displayed by *N* (wrt *A*) if and only if *g*(*T*) is a most parsimonious tree displayed by $N'$ (wrt $A'$).

### Proof

To obtain $N'$ from *N* we split each taxon $x_i$ into a cherry $\{x^1_i$, $x^2_i\}$. If, for a given character, $x_i$ had state 0 (respectively, 1), we give both $x^1_i$ and $x^2_i$ the state 0 (respectively, 1). If $x_i$ had state “-” we give $x^1_i$ state 0 and $x^2_i$ state 1. The idea is that by encoding a gap symbol as a $\{0,1\}$ cherry a single mutation is unavoidably incurred (on one of the two edges leading into $x^1_i$ and $x^2_i$) and thus the state of the parent of the cherry in any (optimal) extension is irrelevant. The parent thus simulates the original gap symbol: the bottom-up phase of Fitch’s algorithm will always allocate the subset of states $\{0,1\}$ to the parent. (The bijection *g*, and its inverse, are trivially computable in polynomial time by splitting each taxon into a cherry, or collapsing cherries, respectively). $\square$

We defer preliminaries relating to likelihood until Sect. 4.

Let *G* be an undirected graph. An *orientation* of *G* is a directed graph $G'$ obtained by replacing each edge $\{u,v\}$ of *G* with exactly one of the two arcs (*u*, *v*) or (*v*, *u*). Given an orientation $G'$ of *G*, a *source* is a node that has only outgoing arcs, and a *sink* is a node that has only incoming arcs. Let *msso*(*G*) denote the maximum, ranging over all possible orientations $G'$ of *G*, of the sum of the number of sources and sinks in $G'$. MAX-SOURCE-SINKS-ORIENTATION is the problem of computing *msso*(*G*). A cubic graph is a graph where every node has degree 3.

The proofs of the following are deferred to the appendix. These two results form the foundation of the hardness results given in the next section.

### Lemma 1

MAX-SOURCE-SINKS-ORIENTATION is NP-hard on cubic graphs.

### Corollary 1

MAX-SOURCE-SINKS-ORIENTATION is APX-hard on cubic graphs.

## Hardness of finding a most parsimonious tree displayed by a network

In this section we will build on Lemma 1 and Corollary 1 to prove that computing a most parsimonious tree displayed by a rooted phylogenetic network *N* with respect to an alignment *A* is NP-hard and APX-hard already for highly restricted instances.

### Theorem 1

It is NP-hard to compute a most parsimonious tree displayed by a rooted phylogenetic network *N* with respect to an alignment *A*, even when *N* is a binary level-1 network with at most 3 outgoing arcs per gall and *A* consists only of two states $\{0,1\}$ and does not contain gap symbols.

### Proof

Let $G = (V,E)$ be a cubic instance of MAX-SOURCE-SINKS-ORIENTATION. We will start by building a binary level-1 network *N* with 6|*E*| taxa and 2|*E*| reticulations, and an alignment *A* on states $\{0,1,\text {``-''}\}$ consisting of 6|*E*| sequences, each sequence of length |*V*|. (We will remove the “-” symbols later). One can thus view *A* as a $\{0,1,-\}$ matrix with 6|*E*| rows and |*V*| columns, or equivalently as a set of |*V*| characters for the 6|*E*| taxa of *N*.

**Fig. 1:**
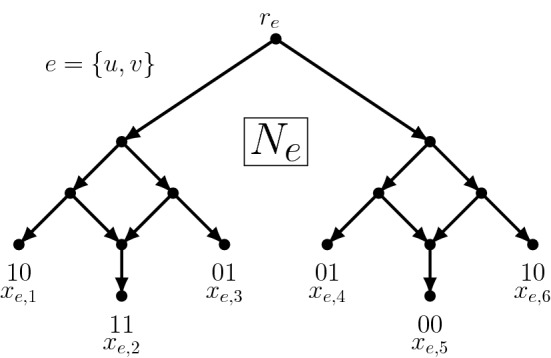
Although each sequence has length |*V*|, only columns *u* and *v* are shown. For this edge, the other $|V|-2$ symbols are “-”

To construct *N*, we start by taking a rooted binary caterpillar on |*E*| taxa. For each $e \in E$ replace the taxon $x_e$ of the caterpillar with a copy $N_e$ of the network shown in Fig. 1. The 6 taxa within $N_e$ are denoted $x_{e,i}$, $i \in \{1,\ldots ,6\}$. We use $r_e$ to refer to the root of $N_e$.

To construct the alignment, we write $$A_{e,i}$$$$(e \in E, i \in \{1, \ldots 6\})$$ to refer to the sequences, and write $$A_{e,i,v}$$ to refer to the state in its *v*th column. These states are assigned as follows. For each edge $$e = \{u,v\} \in E$$, we set the states of the 6 taxa $$A_{e,i,u}$$ ($$i \in \{1, \ldots , 6\}$$) to be 1, 1, 0, 0, 0, 1, the states of the 6 taxa $$A_{e,i,v}$$ ($$i \in \{1, \ldots , 6\}$$) to be 0, 1, 1, 1, 0, 0, and for each $$w \not \in \{u,v\}$$, we set the states of the 6 taxa $$A_{e,i,w}$$ ($$i \in \{1, \ldots , 6\}$$) to all be “-”. Given that each edge is incident to exactly 3 edges, there are exactly $$k := 6|V|(|E|-3)$$ “-” symbols in *A*.

**Fig. 2:**
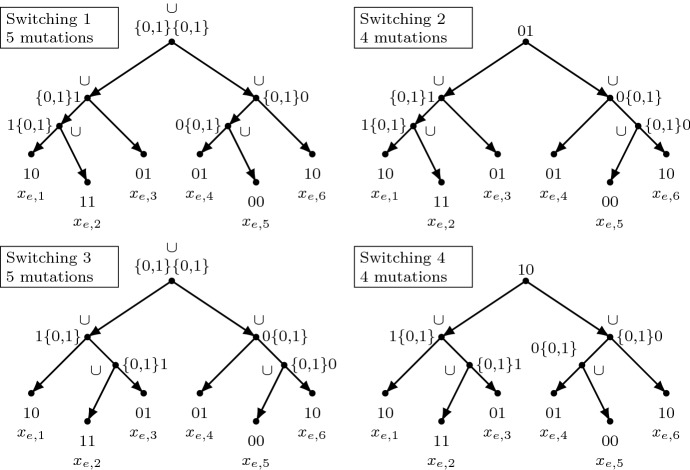
The four switchings possible for $N_e$. The interior nodes are labelled by the output of the bottom-up phase of Fitch’s algorithm, for the two characters concerned. The $\cup$ symbol denotes where union events occur (i.e. mutations are incurred). The critical point is that both switching 2 and 4 incur the fewest number of mutations, and these select for 01 and 10 at the root, respectively, representing the choice of which way to orient edge *e*

Given that each $N_e$ contains 2 reticulations, there are $2^2 = 4$ different switchings of these reticulations possible, shown in Fig. 2. Note that switchings 1 and 3 both induce 5 mutations, while switchings 2 and 4 both induce 4 mutations. (Here by “induce mutations” we are referring to properties (i) and (ii) of Fitch’s algorithm, described in the appendix). We now claim that there exists an optimum solution in which only switchings 2 and 4 are used. Suppose, for some $e = \{u,v\} \in E$, switching 1 or 3 is used. Let *T* be the tree induced by this switching. Fix any optimal extension of *A* to *T*. Let $T_e$ be the subtree of *T* rooted at $r_e$; at least 5 mutations will be incurred on the edges of $T_e$ (with respect to the extension; see property (i) of Fitch’s algorithm). Consider now the states allocated to $r_e$ in columns *u* and *v*. There are four such *uv* combinations: 00, 01, 10, 11. If it is combination 01 or 10, we could replace $T_e$ with the subtree corresponding to switching 2 or 4 (respectively). This replacement subtree incurs only 4 mutations on its edges, so the total number of mutations in *T* decreases. If it is combination 00 or 11 we can use switching 2. This might induce a new mutation (on the edge incoming to $r_e$) but we again save at least one mutation on the edges of the subtree (because at most 4, rather than at least 5 mutations are incurred there), so the overall number of mutations does not increase. Summarizing, whichever combination 00, 01, 10, 11 occurs at $r_e$, we can replace it with switching 2 or 4 without increasing the total number of mutations. Iterating this procedure proves the claim. Henceforth we can thus assume that for each $e \in E$ either switching 2 or 4 is used.

Observe that if, for a given $e = \{u,v\}$, the network $N_e$ uses switching 2, the bottom-up phase of Fitch’s algorithm will allocate 01 (in columns *u* and *v*) to $r_e$. If, on the other hand, switching 4 is used, Fitch’s algorithm will allocate 10. In both cases, exactly 4 union events are generated on the nodes (of the subtree of $N_e$ induced by the switching). See Fig. 2 for elucidation.

The central idea is that, since, for an edge $e=\{u,v\}$, a state 0 (resp. 1) in *v* implies a state 1 (resp. 0) in *u* and vice versa, we can use the choice of whether to use switching 2 or 4 (for each of the |*E*| reticulation pairs) to encode a choice as to which way to orient the corresponding edge. Without loss of generality we use state 0 to denote incoming edges, and state 1 to denote outgoing edges. Consider the bottom-up phase of Fitch’s algorithm. Observe that, if a vertex *v* incident to three edges $e_1, e_2, e_3$ becomes a *sink*, the states at the roots of $N_{e_1}, N_{e_2}, N_{e_3}$ (in column *v*) will *all* be 0, and for each $e' \not \in \{e_1, e_2, e_3\}$ the states at the root of $N_{e'}$ (in column *v*) will be “-” i.e. “don’t care”^2^. Continuing Fitch’s algorithm along the backbone of the caterpillar shows that no mutations will be incurred on the edges of the caterpillar in column *v*. A completely symmetrical situation holds if a vertex becomes a *source*: the states at the roots of $N_{e_1}, N_{e_2}, N_{e_3}$ (in column *v*) will *all* be 1, and again no mutations are incurred on the edges of the caterpillar. On the other hand, if a vertex *v* is neither a source nor a sink, then the states assigned by the bottom-up phase of Fitch’s algorithm to the roots of $N_{e_1}, N_{e_2}, N_{e_3}$ (in column *v*) will consist of 0 (twice) and 1 (once) or 1 (twice) and 0 (once). Either way exactly 1 mutation is then incurred on the edges of the caterpillar (as can be observed by running the top-down phase of Fitch’s algorithm).

This means that the parsimony score is minimized by creating as many sources and sinks as possible. Specifically we have

$$\begin{aligned} l_A(N) = 6|V| + (|V| - msso(G)). \end{aligned}$$

Each edge in the graph will induce 4 mutations (within the $N_e$ part), and $|E| = 3|V|/2$, which explains the term 6|*V*|. As argued above, sources and sinks to do not increase the parsimony score, and all other vertices increase the parsimony score by exactly 1, hence the term $(|V| - msso(G))$.

Clearly *msso*(*G*) can easily be calculated from $l_A(N)$. Finally, we can apply Observation 1 to obtain a network $N'$ and $A'$ without “-” symbols such that

$$\begin{aligned} l_{A'}(N') = 6|V|(|E|-3) + 6|V| + (|V| - msso(G)) \end{aligned}$$

The transformation does not raise the level of the network or the number of arcs outgoing from any biconnected component. NP-hardness follows. $\square$

If we *do* allow “-” symbols then the following slightly stronger result is obtained: APX-hardness implies NP-hardness but additionally excludes the existence of a Polynomial Time Approximation Scheme (PTAS), unless P=NP. APX-hardness does not obviously hold if we encode the gap symbols using Observation 1 because the additive *O*(|*V*||*E*|) term thus created distorts the objective function.

### Corollary 2

It is APX-hard to compute a most parsimonious tree displayed by a rooted phylogenetic network *N* with respect to an alignment *A*, even when *N* is a binary level-1 network with at most 3 outgoing arcs per gall and *A* consists only of states $\{0,1,\text {``-''}\}$.

### Proof

We give a (14, 1) L-reduction from *msso*, which is APX-hard, to the parsimony problem. L-reductions preserve APX-hardness so the result will follow. An $(\alpha , \beta )$ L-reduction (Papadimitriou and Yannakakis 1991), where $\alpha , \beta \ge 0$, is defined as follows.

### Definition 1

Let *A*, *B* be two optimization problems and $c_A$ and $c_B$ their respective cost functions. A pair of functions *f*, *g*, both computable in polynomial time, constitute an $(\alpha , \beta )$ L-reduction from *A* to *B* if the following conditions are true:

1. For every instance *x* of *A*, *f*(*x*) is an instance of *B*,
2. For every feasible solution *y* of *f*(*x*), *g*(*y*) is a feasible solution of *x*,
3. For every instance *x* of *A*, $OPT_B(f(x)) \le \alpha OPT_A(x)$,
4. For every feasible solution $y'$ of *f*(*x*) we have $|OPT_A(x) - c_A(g(y'))| \le \beta |OPT_B(f(x)) - c_B(y')|$

where $OPT_{A}$ is the optimal solution value of problem *A* and similarly for *B*.

For brevity we refer to the optimum size of the parsimony problem as *mp*(*N*, *A*). We use the reduction described in the proof of Theorem 1 (before the gap symbols have been removed) with some slight modifications. The forward-mapping function *f* (condition 1 of the L-reduction) is the same mapping used in the proof of Theorem 1. The back-mapping function *g* (i.e. condition 2) will be described below. To establish condition 3 for a given $(\alpha , \beta )$ we need to prove that $mp(N,A) \le \alpha \cdot msso(G)$. Now, we know that $msso(G) = maxcut(G)-|V|/2$ (see appendix) and that $maxcut(G) \ge 2/3 |E| = |V|$ (because every cubic graph has a cut at least this large simply by moving nodes which have more neighbours on their side of the cut, to the other side). Hence, $msso(G) \ge |V|/2$. We know that $mp(N,A) = 7|V| - msso(G)$. Trivially therefore $mp(N,A) \le 7|V|$. Hence taking $\alpha =14$ is sufficient. For the other direction, we need to show that for an arbitrary solution to the parsimony problem, which induces *p* mutations, the back-mapping function yields an orientation of *G* with *s* sources and sinks such that $|msso(G)-s| \le \beta |p-mp(N,A)|$. The back-mapping function *g* first ensures that all the $N_e$ gadgets are using type 2 or type 4 switchings, which might reduce the number of mutations to $p' \le p$, and then extracts an orientation of *G* (thus establishing condition 2). Now, $s = 7|V|-p'$ and $mp(N,A) = 7|V|-msso(G)$ so

$$\begin{aligned} msso(G) - s&= msso(G) - (7|V|-p')\\&= msso(G) - 7|V| + p'\\&= p' - (7|V| - msso(G))\\&= p' - mp(N,A)\\&\le p - mp(N,A). \end{aligned}$$

So taking $\beta = 1$ is sufficient to establish condition 4. $\square$

## Hardness of finding a most likely tree displayed by a network

*The likelihood of a tree*. We now introduce the basic concepts and notation that are necessary to define the likelihood of a tree with respect to an alignment. We largely follow the simple formulation by Roch (2006). First, we need a probabilistic model describing how sequences evolve along a tree. Here we assume the simplest model available, known as the Cavender-Farris model (Farris 1973; Cavender 1978), which can be described as follows. Let $T = (V,E)$ be a rooted binary phylogenetic tree on *X*. We associate probabilities $\mathbf {p}=(p_e)_{e\in E} \in [0,\nicefrac {1}{2}]^{|E|}$ to the edges of *T* and denote this $(T,\mathbf {p})$. Under the Cavender-Farris model, each character evolves independently, as follows: at the root pick randomly a state between 0 and 1, each with probability $\nicefrac {1}{2}$, and then, for each vertex *v* below the root, either copy the state of the parent of *v* or flip it, with probabilities $1-p_e$ and $p_e$, respectively. The restriction $p_e\le \nicefrac {1}{2}$ corresponds to the fact that, in a symmetric model, no amount of time can make a character more likely to change state than to remain in the same state.

The process described above eventually associates a state to each element of *X* at the leaves of the tree, that is, it generates a random binary character. The probability of generating the binary character *f* is called the *likelihood* of $(T,\mathbf {p})$ with respect to *f*, denoted $L_f(T,\mathbf {p})$, and can be calculated as follows:

$$\begin{aligned} L_f(T,\mathbf {p}) = \sum _{\hat{f}} \frac{1}{2} \prod _{e = (u,v) \in E} p_e^{|\hat{f}(v)-\hat{f}(u)|} (1-p_e)^{1-|\hat{f}(v)-\hat{f}(u)|} \end{aligned}$$

Here $\hat{f}$ ranges over all extensions of *f* to *T*. Because the model assumes that the characters in a sequence evolve independently, the probability of generating the binary sequences in an alignment *A*, named the *likelihood* of $(T,\mathbf {p})$ with respect to *A*, denoted $L_A(T,\mathbf {p})$, can be obtained as

$$\begin{aligned} L_A(T,\mathbf {p}) = \prod _{f \in A} L_f(T,\mathbf {p}) \end{aligned}$$

(Here, and in the rest of this section, we assume that alignments do not contain gap symbols.)

We now introduce some more notation that will be useful in the following. An *extension*$\widehat{A}$ of an alignment *A* to a tree $T=(V,E)$ is a set of functions $\hat{f}: V \rightarrow \{0,1\}$ obtained by taking exactly one extension of each character in *A*. In practice, $\widehat{A}$ can be represented as a matrix with |*V*| rows and |*A*| columns, in which the rows corresponding to the leaves of *T* are identical to the rows of *A*. For $e=(u,v) \in E$, we denote by $h_e(\widehat{A})$ the number of differences (that is, the Hamming distance) between the sequences that $\widehat{A}$ associates to *u* and *v*. Finally, let $l_{\widehat{A}}(T)$ denote $\sum _{e\in E} h_{e}(\widehat{A}) = \sum _{\hat{f}\in \widehat{A}} l_{\hat{f}}(T)$. Note that the parsimony score $l_{A}(T)$ is the minimum of $l_{\widehat{A}}(T)$ over all extensions of *A*. Given these notations, we can express the likelihood of $(T,\mathbf {p})$ as follows, where $m=|A|=|\widehat{A}|$, and $\widehat{A}$ ranges over all extensions of *A*:

1 $$\begin{aligned} L_A(T,\mathbf {p}) = \sum _{\widehat{A}} 2^{-m}\prod _{e \in E} p_e^{h_{e}(\widehat{A})} (1-p_e)^{m-h_{e}(\widehat{A})} \end{aligned}$$

Each term in the sum in Eqn. (1) expresses the product of the probabilities of transition between the sequences associated by $\widehat{A}$ to the endpoints of the edges, times the probability of the sequence at the root.

**Fig. 3:**
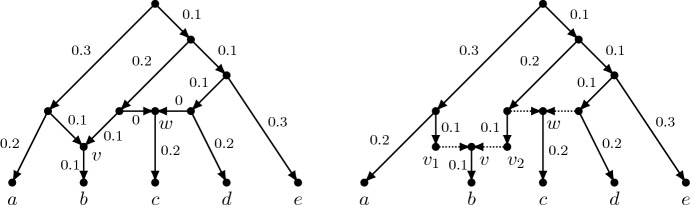
Alternative representations of a phylogenetic network having some reticulation edges with strictly positive lengths. A reticulation edge with positive length should be interpreted as ending in a node that undergoes a reticulation event, but leaves no descendant in the network, other than the reticulation node itself. Both edges entering node *v* in the network on the left are an example of this. The representation on the right, which strictly speaking is not a phylogenetic network, makes the biological interpretation of these edges explicit. In this representation, dashed edges denote an instantaneous event, and their length is necessarily 0 (not shown)

*Networks with edge probabilities*. It is possible to extend the Cavender-Farris model to describe the evolution of binary sequences on a binary rooted network *N*. For every edge *e* of *N*, we define a probability $p_e'\in [0,\nicefrac {1}{2}]$ which represents again the probability of change between 0 and 1 along edge *e*. The evolution of a single character follows the same rules as in the case of a tree, except that when setting the state at a reticulation vertex *v*, one of its two parents is randomly selected with a probability $\gamma _v \in (0,1)$ (and $1-\gamma _v$ for the other parent), also given as a parameter of the model. The state at *v* is generated as if the selected parent of *v* were the only parent of *v*, as in the tree case. That includes taking into account the probability of change $p_e'$ along the edge connecting the selected parent to *v*. The inheritance probability parameters $\gamma _v$ (e.g. Yu et al. 2012; Wen et al. 2016; Zhang et al. 2017), and mechanisms to correlate inheritance between neighboring characters in a sequence will not be discussed in the remainder of this paper. These aspects of the model are necessary to define the likelihood of the network, but they are irrelevant for the likelihood of the trees displayed by the network, which is all that concerns us here. In the following, we denote a network *N* and the probabilities of change along its edges as $(N,\mathbf {p'})$.

We note that in some cases, the edges entering a reticulation node (the *reticulation edges*) may represent an event of instantaneous combination between the sequences at the tails of the reticulation edges. The probabilities of change $p_e'$ for these reticulation edges will necessarily equal 0, as they represent an immediate transfer of genetic information, and there is no time for sequence changes along these edges. The edges entering node *w* in the network of Fig. 3 are an example of this. Not all edges entering a reticulation, however, need be of this type. For the sake of generality, the networks we consider here may have “non-istantaneous” reticulation edges, that is reticulation edges with $p_e'>0$. For example, consider the edges entering node *v* in the network of Fig. 3. Edges like these simply mean that before the reticulate event happened, the sequence in the edge leading to the reticulation evolved independently of the rest of the tree, potentially accumulating changes. At the end of the edge, the sequence that underwent the reticulate event left no descendant leading to a leaf, other than the sequence at the reticulation itself. Figure 3 also displays a different representation of the same network, showing separately the nodes $v_1$ and $v_2$ that underwent the reticulation event. Neither of these nodes left any other descendant than *v* within the network. In some biological contexts (for example when reticulations represent homologous recombinations), reticulation edges representing lineages that have existed for a strictly positive amount of time are the norm, and not the exception. Examples of this are the phylogenetic networks generated by the coalescent with recombination model (e.g. Griffiths and Marjoram 1997; Nordborg 2001), or by the birth-hybridization process (Zhang et al. 2017) where reticulate edges of zero length are practically impossible.

*Trees displayed by a network with edge probabilities*. We say that a network $(N,\mathbf {p'})$ displays a tree $(T,\mathbf {p})$, if *N* displays *T* in the usual topological sense (i.e. some subdivision $T'$ of *T* is a subtree of *N*) *and*$(T,\mathbf {p})$ can be obtained from $T'$ by repeatedly suppressing vertices with indegree-1 and outdegree-1, where here suppression also updates the probabilities. Specifically, if $T'$ contains two edges $e_1 = (u,v)$ and $e_2 = (v,w)$, where *v* has indegree-1 and outdegree-1, the suppression operation replaces these two edges with a single edge $e = (u,w)$ and assigns it the probability

2 $$\begin{aligned} p_e = p_{e_1}(1-p_{e_2}) + (1-p_{e_1})p_{e_2}. \end{aligned}$$

This expresses the probability of having different states at the endpoints of a two-edge path, under the Cavender-Farris model.

**Fig. 4:**
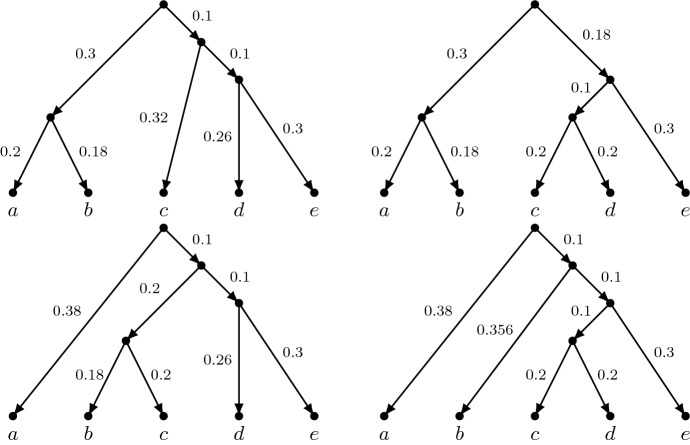
The four trees with edge probabilities displayed by the network in Fig. 3. Note that the edge probability 0.356 in the fourth network is obtained by two applications of Eqn. (2)

It is important to observe that, as a result of the definitions above, if a network $(N,\mathbf {p'})$ describes the evolution of a set of characters, then any of these characters taken separately evolves according to the Cavender-Farris model for one of the trees $(T,\mathbf {p})$ displayed by $(N,\mathbf {p'})$. Figure 4 shows, as an example, the four trees displayed by the network in Fig. 3.

Note that, in general, a tree *T* can have multiple distinct images $T'$ in the network, so it can occur that $(N,\mathbf {p'})$ displays $(T,\mathbf {p})$ for multiple different $\mathbf {p}$. Also note that because $p_e'\le \nicefrac {1}{2}$ for all edges in the network, the same will hold for the edges of the trees it displays, as no repeated application of Eqn. (2) can produce a probability $p_e>\nicefrac {1}{2}$ from edge probabilities that are at most $\nicefrac {1}{2}$. It is also easy to see that if $0< p_{e_1}, p_{e_2}< \nicefrac {1}{2}$, then $\max \{p_{e_1},p_{e_2}\}< p_e < p_{e_1}+p_{e_2}$. These observations lead to the following one, which will be useful later on:

### Observation 2

Let $(N,\mathbf {p'})$ be such that for every edge of *N*, $0<p'_e<\nicefrac {1}{2}$. Let $(T,\mathbf {p})$ be a tree displayed by $(N,\mathbf {p'})$ and *e* an edge of *T*. Finally, let $E'(e)$ be the subset of the edges of *N* whose probabilities contribute to $p_e$. Then, $p_e < \nicefrac {1}{2}$ and

$$\begin{aligned} \max _{e'\in E'(e)} p_{e'}< p_e < \sum _{e'\in E'(e)} p_{e'} \end{aligned}$$

We say that $(T^*,\mathbf {p^*})$ is a *most likely (ML) tree displayed by*$(N,\mathbf {p'})$*(with respect toA*) if it maximizes $L_A(T,\mathbf {p})$, ranging over all $(T, \mathbf {p})$ displayed by $(N,\mathbf {p'})$. In the remainder of this section we consider the problem of finding such a most likely tree given a network with edge probabilities and an alignment.

*A link between likelihood and parsimony*. There are well-known relationships between the likelihood and the parsimony of a tree that imply that under some conditions a most likely tree is also a most parsimonious one (Tuffley and Steel 1997). We now illustrate one such relationship (Corollary 3 below), which is based on the observation that as we reduce the scale of a tree, its likelihood converges to zero at a rate that only depends on its parsimony score. Although it shares similarities with the results by Tuffley and Steel, we are not aware that it has been explicitly stated in the literature. This result is *not* necessary to obtain the other results in this section, but it provides the intuition behind them.

In the following statements, we assume that $c\in (0,1)$, so the form $c\rightarrow 0$ is to be understood as *c* approaches 0 to the right. Also, $c\mathbf {p}$ simply denotes the product between the scalar *c* and vector $\mathbf {p}$.

### Lemma 2

The function $f(c) = L_A(T,c\mathbf {p})$ is $\Theta (c^{l_A(T)})$ as $c\rightarrow 0$.

### Proof

Write $L_A(T,c\mathbf {p})$ using Eqn. (1):

$$\begin{aligned} L_A(T,c\mathbf {p})&= \sum _{\widehat{A}} 2^{-m} \prod _{e \in E} (cp_e)^{h_{e}(\widehat{A})} (1-cp_e)^{m-h_{e}(\widehat{A})} \\&= \sum _{\widehat{A}} 2^{-m}c^{l_{\widehat{A}}(T)}\cdot \prod _{e \in E} p_e^{h_{e}(\widehat{A})} (1-cp_e)^{m-h_{e}(\widehat{A})}, \end{aligned}$$

where we have used $\sum _{e\in E} h_{e}(\widehat{A})= l_{\widehat{A}}(T)$. Note that the products in the second expression tend to a constant as $c\rightarrow 0$. As a consequence, the term for $\widehat{A}$ in the sum has order $\Theta (c^{l_{\widehat{A}}(T)})$ as $c\rightarrow 0$. Since the lowest degree dominates, their sum is $\Theta (c^{l_A(T)})$. $\square$

### Corollary 3

Let *A* be an alignment and $T_1$ and $T_2$ two trees such that $l_A(T_1) < l_A(T_2)$. Then, for any $\mathbf {p_1}$ and $\mathbf {p_2}$,

$$\begin{aligned} L_A(T_1,c\mathbf {p_1}) > L_A(T_2,c\mathbf {p_2})\quad \text {for }c \text { sufficiently close to 0.} \end{aligned}$$

### Proof

As $c\rightarrow 0$, $L_A(T_1,c\mathbf {p_1})$ is $\Theta (c^{l_A(T_1)})$, while $L_A(T_2,c\mathbf {p_2})$ is $\Theta (c^{l_A(T_2)})$. That is, $L_A(T_1,c\mathbf {p_1})$ converges to 0 at a lower rate than $L_A(T_2,c\mathbf {p_2})$. Thus there exists a neighborhood of 0 in which $L_A(T_1,c\mathbf {p_1}) > L_A(T_2,c\mathbf {p_2})$ holds. $\square$

The corollary above can be extended to any collection of trees: irrespective of the edge probabilities assigned to them, if the trees are rescaled by a sufficiently small *c*, the most parsimonious trees will have likelihoods greater than all the other trees, meaning that a most likely tree in the collection of rescaled trees will necessarily also be most parsimonious.

*Proving the NP-hardness of finding an ML tree in a network*. In the remainder of this section, namely in the statements of the next two formal results, we are implicitly given a network *N* on *X* with $|X|=n$ and an alignment *A* with *m* characters on *X*. The *height* of a network *N* is the maximum number of edges in a directed path in *N*.

### Lemma 3

Let $(N,\mathbf {c})$ be a network of height $d_N$, where all the edges are assigned a constant probability $c_e=c$, with $0<c<\nicefrac {1}{2}$. Let $(T,\mathbf {p})$ be a tree displayed by $(N,\mathbf {c})$. Then,

$$\begin{aligned} 2^{-2mn} \cdot c^{l_A(T)}< L_A(T,\mathbf {p}) < 2^{mn} \cdot d_N^{2mn} \cdot c^{l_A(T)} \end{aligned}$$

### Proof

Using Observation 2, we note that, for any edge $e\in E$ of $T=(V,E)$, $c<p_e<\min \{cd_N,\nicefrac {1}{2}\}$.

We begin by proving the upper bound in the statement. From Eqn. (1) and the fact that $(1-p_e)^{m-h_{e}(\widehat{A})} <1$, we get the first inequality in the following:

$$\begin{aligned} L_A(T,\mathbf {p})&< \sum _{\widehat{A}} 2^{-m}\prod _{e \in E} (cd_N)^{h_{e}(\widehat{A})} \\&= \sum _{\widehat{A}} 2^{-m} d_N^{l_{\widehat{A}}(T)} c^{l_{\widehat{A}}(T)} \quad < 2^{m(n-1)-m} d_N^{m(2n-2)} c^{l_A(T)}, \end{aligned}$$

where the last inequality is obtained by noting that the sum has $2^{m(n-1)}$ terms (there are $n-1$ internal nodes in a rooted binary tree, and thus $2^{m(n-1)}$ different extensions of *A*), and that $l_A(T) \le l_{\widehat{A}}(T) \le m(2n-2)$ (there are $2n-2$ branches in a rooted binary tree, and thus we cannot have more than $2n-2$ changes per character). The upper bound in the statement is larger than the one above.

As for the lower bound, if we use $c<p_e<\nicefrac {1}{2}$ in Eqn. (1):

$$\begin{aligned} L_A(T,\mathbf {p})&> \sum _{\widehat{A}} 2^{-m}\prod _{e \in E} c^{h_{e}(\widehat{A})} {1/2}^m\\&= \sum _{\widehat{A}} 2^{-m} c^{l_{\widehat{A}}(T)} 2^{-m(2n-2)} \quad > 2^{-m(2n-1)} c^{l_A(T)}, \end{aligned}$$

where the last inequality is obtained by taking only one term in the sum. The lower bound in the statement is smaller than the one above. $\square$

The lemma above shows the order of convergence to 0 of the likelihood $L_A(T,\mathbf {p})$ of a tree displayed by $(N,\mathbf {c})$ as $c\rightarrow 0$. The higher the parsimony score, the faster the convergence. As a consequence, for *c* sufficiently close to 0, a tree with a lower parsimony score than another will have a higher likelihood. The following lemma shows how close is “sufficiently close”, by providing an explicit upper bound to *c*.

### Proposition 1

Let $(N,\mathbf {c})$ be a network of height $d_N$, where all the edges are assigned a constant probability $c_e=c$, with $0<c<d_N^{-2mn}2^{-3mn}$. If $(T^*,\mathbf {p^*})$ is a most likely tree displayed by $(N,\mathbf {c})$, then $T^*$ is a most parsimonious tree displayed by *N*. $\square$

### Proof

Suppose that $(T^*,\mathbf {p^*})$ is a most likely tree displayed by $(N,\mathbf {c})$, but not most parsimonious. That is, there exists $(T,\mathbf {p})$ displayed by $(N,\mathbf {c})$ with $l_A(T)\le l_A(T^*)-1$. But then, by using the lower bound in Lemma 3:

$$\begin{aligned} L_A(T,\mathbf {p}) > 2^{-2mn} \cdot c^{l_A(T)} \ge 2^{-2mn} \cdot c^{l_A(T^*)-1}. \end{aligned}$$

Now apply the upper bound in Lemma 3 to $T^*$, and combine it with $c<d_N^{-2mn}2^{-3mn}$:

$$\begin{aligned} L_A(T^*,\mathbf {p^*})< & {} 2^{mn} \cdot d_N^{2mn} \cdot c^{l_A(T^*)} < 2^{mn} \cdot d_N^{2mn} \cdot c^{l_A(T^*)-1} \cdot d_N^{-2mn} \cdot 2^{-3mn} \\= & {} 2^{-2mn} \cdot c^{l_A(T^*)-1} \end{aligned}$$

The last terms of the two chains of inequalities above are equal, thus proving $L_A(T,\mathbf {p})>L_A(T^*,\mathbf {p^*})$. Since this contradicts the assumption that $(T^*,\mathbf {p^*})$ is a most likely tree, the statement follows. $\square$

The proposition above shows that the NP-hard problem of finding a most parsimonious tree in a network *N* with respect to an alignment *A* can be reduced to the problem of finding a most likely tree in $(N,\mathbf {c})$ with respect to *A*, where $\mathbf {c}=(c_e)$ is such that $c_e=c$, and $0<c<d_N^{-2mn}2^{-3mn}$. Since the reduction preserves the network and the alignment, the main result of this section follows from Theorem 1:

### Theorem 2

It is NP-hard to compute a most likely tree $(T,\mathbf {p})$ displayed by a rooted phylogenetic network $(N,\mathbf {p'})$ with respect to an alignment *A*, even when *N* is a binary level-1 network with at most 3 outgoing arcs per gall and *A* consists only of two states $\{0,1\}$ and does not contain gap symbols.

## Conclusions and open problems

We have shown that, given a phylogenetic network with a sequence for each leaf, finding a most parsimonious or most likely tree displayed by the network is computationally intractable (NP-hard). Moreover, this is the case even when we restrict to binary sequences and level-1 networks; the simplest networks that are not trees. However, many computational problems that can be shown to be theoretically intractable can be solved reasonably efficiently in practice (see e.g. *Cautionary Tales of Inapproximability* by Budden and Jones (2017)). We end the paper by discussing whether we expect this to be the case for our problem.

There is a dynamic programming algorithm, described in Theorem 5.7 of Fischer et al. (2015), for finding a tree in a network that is most parsimonious with respect to a single character. The running time is fixed-parameter tractable, with as parameter the level of the network. Hence, this algorithm is practical as long as the level of the network is not too large. This algorithm can easily be extended to multiple characters (that all have to choose the same tree) when the number of characters is adopted as a second parameter. Indeed, for every root of a biconnected component, we introduce a dynamic programming entry not just for every possible state but for every possible sequence of states. However, the running time of this algorithm would be exponential in the number of characters, which makes it useless for almost all biological data. Similarly, the Integer Linear Programming (ILP) solution presented in the same paper can also be easily extended to multiple characters. However, there does not seem to be an easy way to do that without having the number of variables growing linearly in the number of characters. Hence, this approach is also unlikely to be useful in practice.

In contrast, consider the simple algorithm that loops through the at most $2^r$ trees displayed by the network, with *r* the number of reticulation nodes in the network, and computes the parsimony or likelihood of each tree (this naïve FPT algorithm was presented in Nakhleh et al. (2005), where it is named *Net2Trees*). Ironically, this simple algorithm would outperform the approaches mentioned above for any kind of data with a reasonably large number of characters. Hence, the main open question that remains is whether there exists an algorithm whose running time is linear (or at least polynomial) in the number of characters and whose dependency on *r* is better than $2^r$ (for example recently an algorithm with exponential base smaller than 2 was discovered for the tree containment problem (Gunawan et al. 2016), although this algorithm does not obviously extend to generating all trees in the network). Another question of interest that remains open is the following: does the parsimony problem under restrictions (1)-(3) listed in the introduction permit good (i.e. constant factor) approximation algorithms, and possibly even a PTAS, when the alignment *A* does not contain any indels?

## Appendix: Fitch’s algorithm

Fitch’s algorithm (Fitch 1971) has two phases. In the first phase, known as the *bottom-up* phase, we start by assigning the singleton subset of states $\{f(x)\}$ to each taxon *x*. The internal nodes of *T* are assigned subsets of states recursively, as follows. Suppose a node *p* has two children *u* and *v*, and the bottom-up phase has already assigned subsets *F*(*u*) and *F*(*v*) to the two children, respectively. If $F(u) \cap F(v) \ne \emptyset$ then set $F(p) = F(u) \cap F(v)$ (in which case we say that *p* is an *intersection* node). If $F(u) \cap F(v) = \emptyset$ then set $F(p) = F(u) \cup F(v)$ (in which case we say that *p* is a *union* node). The number of union nodes in the bottom-up phase is equal to $l_f(T)$. To actually create an optimal extension $\hat{f}$, we require the *top-down* phase of Fitch’s algorithm. Start at the root *r* and let $\hat{f}(r)$ be any element in *F*(*r*). For an internal node *u* with parent *p*, we set $\hat{f}(u) = \hat{f}(p)$ (if $\hat{f}(p) \in F(u)$) and otherwise (i.e. $\hat{f}(p) \not \in F(u)$) set $\hat{f}(u)$ to be an arbitrary element of *F*(*u*).

For each node *u* of the tree, let $\cup (u)$ be the number of union events in the subtree rooted at *u*. The following well-known properties of Fitch’s algorithm are used repeatedly in the main hardness proof of this article: (i) *every* extension (optimal or otherwise) must incur *at least*$\cup (u)$ mutations on the edges of the subtree rooted at *u*; (ii) an extension created by Fitch’s algorithm induces *exactly*$\cup (u)$ mutations on the edges of the subtree rooted at *u* (and *u* is assigned a state from *F*(*u*) in this extension).

## Appendix: NP-hardness and APX-hardness of MAX-SOURCE-SINKS-ORIENTATION

The following result is based on a sketch proof by Colin McQuillan^3^. We have been unable to find an original reference and hence have reconstructed the proof in detail. The APX-hardness proof is original.

### Lemma 1

MAX-SOURCE-SINKS-ORIENTATION is NP-hard on cubic graphs.

### Proof

Recall that the classical MAX-CUT problem asks us to bipartition the vertex set of an undirected graph *G*, such that the number of edges that cross the bipartition is maximized. We reduce from the NP-hard problem CUBIC-MAX-CUT which is the restriction of the MAX-CUT problem to cubic graphs. Given an undirected cubic graph *G*, we simply write *maxcut*(*G*) to denote the number of edges in the maximum-size cut.

We reduce CUBIC-MAX-CUT to MAX-SOURCE-SINKS-ORIENTATION. Specifically, given an undirected cubic graph $G = (V,E)$ (i.e. an instance of CUBIC-MAX-CUT) we will show that $msso(G) =maxcut(G) - |V|/2$, from which the hardness will follow.

We start by proving that $msso(G) \ge maxcut(G) - |V|/2$. Fix an arbitrary cut *C* of *G* and let (*U*, *W*) be the corresponding bipartition. If some vertex of *U* or *W* has more neighbours on the other side of the partition than its own, move it to the other side of the partition: this will increase the size of the cut. We repeat this until it is no longer possible and let *C* and (*U*, *W*) refer to the cut and its induced partition at the end of this process. Note that now each vertex in *U* (respectively, *W*) will have at most one neighbour in *U* (respectively, *W*). We proceed by orienting the edges in the cut from *U* to *W*. Now, the remaining edges are either internal to *U* or internal to *W*. These edges must form a matching (i.e. they are node disjoint). For each such edge in *U* (respectively, *W*), exactly one endpoint will become a source (respectively, sink). Nodes in *U* (respectively, *W*) that are not adjacent to internal edges will already be sources (respectively, sinks) due to the orientation of the cut edges from *U* to *W*. Hence, if we write |*C*| to denote the number of edges in the cut *C*, we obtain an orientation of *G* with at least

$$\begin{aligned} (|E| -|C|) + (|V| - 2(|E|-|C|)) \end{aligned}$$

sources and sinks. Hence,

$$\begin{aligned} msso(G)&\ge (|E| -maxcut(G)) + (|V| - 2(|E|-maxcut(G)))\\&= maxcut(G) + |V| - |E| \\&= maxcut(G) + |V| - (3/2)|V| \\&= maxcut(G) - |V|/2. \end{aligned}$$

For the other direction, fix an arbitrary orientation of *G* and let *s* be the number of sources and sinks created by the orientation. We write $V_i$ ($i \in \{0,1,2,3\}$) to denote those vertices of *G* which have indegree *i*. Let $U = V_0 \cup V_1$ and let $W = V_2 \cup V_3$. Whenever an edge of *G* has been oriented from *W* to *U*, reverse its orientation: this only decreases the indegrees of the vertices in *U* and increases the indegrees of vertices in *W* so it cannot destroy any sources or sinks and it cannot cause a node to be on the “wrong” side of the bipartition. (In fact, it will cause the number of sources and sinks to increase, so this situation can only occur if the orientation was not optimal). Let *s* now refer to the number of sources and sinks once all arcs have been oriented from *U* to *W*. The edges (*u*, *w*) such that $u \in U$ and $w \in W$ form a cut; it remains only to count how many of these edges there are. We first count from the perspective of the vertices in *U*. The nodes in $V_0$ each generate 3 outgoing arcs. Let *k* be the total number of edges of the form $(u_0, u_1)$ where $u_0 \in V_0$ and $u_1 \in V_1$. Note that each node in $V_1$ that does not receive any of these *k* arcs, must receive an arc which is outgoing from some other node in $V_1$. It follows that the number of edges in the cut is

$$\begin{aligned} (3|V_0| - k) + (2|V_1| - (|V_1|-k)) = 3|V_0| + |V_1|. \end{aligned}$$

If we count in a symmetrical fashion from the perspective of *W*, and let $\ell$ be the number of arcs of the form $(u_2, u_3)$ where $u_2 \in V_2$ and $u_3 \in V_3$, it follows that the number of edges in the cut is

$$\begin{aligned} (3|V_3| - \ell ) + (2|V_2| - (|V_2| - \ell )) = 3|V_3| + |V_2|. \end{aligned}$$

If we sum these two equations, we obtain a cut with at least the following number of edges:

$$\begin{aligned}&\ge (3/2)( |V_0| + |V_3| ) + (1/2)( |V_1| + |V_2| ) \\&= (3/2) s + (1/2)(|V| - s)\\&= s + (1/2)|V|. \end{aligned}$$

From this follows that $msso(G) \le maxcut(G) - (1/2)|V|$. $\square$

The hardness of *msso* can be strengthened to the following inapproximability result. Note that one consequence of APX-hardness is that *msso* does not permit a PTAS, unless $P=NP$.

### Corollary 1

MAX-SOURCE-SINKS-ORIENTATION is APX-hard on cubic graphs.

### Proof

Note that the constructions and transformations used in the proof of Lemma 1 are all constructive and can easily be conducted in polynomial time. Moreover, they apply to arbitrary cuts/orientations, and not just optimal ones. This allows us to easily strengthen the described reduction to obtain a (1, 1) L-reduction from CUBIC-MAX-CUT to MAX-SOURCE-SINKS-ORIENTATION (see the main text for the definition of L-reduction). From this APX-hardness will follow, since CUBIC-MAX-CUT is APX-hard (Alimonti and Kann 1997; Berman and Karpinski 1999) and L-reductions are APX-hardness preserving. The (1, 1) means that the inapproximability threshhold for MAX-SOURCE-SINKS-ORIENTATION is at least as strong as that for CUBIC-MAX-CUT.

Let $G = (V,E)$ be an instance of CUBIC-MAX-CUT. The forward mapping function *f* (from instances of CUBIC-MAX-CUT to MAX-SOURCE-SINKS-ORIENTATION) is simply the identity function. To be an $(\alpha ,\beta )$ L-reduction, where $\alpha ,\beta \ge 0$, we first have to show that $msso( f(G) ) = msso( G ) \le \alpha \cdot maxcut(G)$. We know that $maxcut(G) - (1/2)|V| = msso(G)$, so $\alpha = 1$ is trivially satisfied. We next have to show a polynomial-time computable back-mapping function *g* (from feasible solutions of MAX-SOURCE-SINKS-ORIENTATION to feasible solutions of CUBIC-MAX-CUT) with the following property: an orientation that induces *s* sources and sinks is mapped to a cut with *k* edges such that $|maxcut(G)-k| \le \beta |msso(G) - s|$. The function *g* was already implicitly described in the NP-hardness reduction: reverse any edges oriented from *W* to *U* (which possibly increases the number of sources and sinks to $s' \ge s$) and extract a cut of size $s' + |V|/2$. Observe,

$$\begin{aligned} maxcut(G) - (s' +|V|/2)&\le maxcut(G) - (s +|V|/2)\\&\le (msso(G)+|V|/2) - (s+|V|/2)\\&\le msso(G) -s. \end{aligned}$$

So taking $\beta =1$ is sufficient. $\square$

## Footnotes

1 Two other definitions of the parsimony score of a network exist in the literature: the *hardwired* parsimony score (Kannan and Wheeler 2012) and parental parsimony score (van Iersel et al. 2018); see the latter manuscript for a discussion about the differences of these three models.

2 Fitch’s algorithm is not well-defined on “-” symbols, but the intuition is that it behaves exactly like the subset of states $\{0,1\}$ behaves in Fitch’s algorithm. This, in fact, is exactly how the construction described in Observation 1 removes “-” symbols from the alignment.

## References

[CR1] Abbott R, Albach D, Ansell S, Arntzen JW, Baird SJ, Bierne N, Boughman J, Brelsford A, Buerkle CA, Buggs R (2013). Hybridization and speciation. J Evol Biol.

[CR2] Alimonti P, Kann V (1997) Hardness of approximating problems on cubic graphs. In: Italian conference on algorithms and complexity (CIAC), pp 288–298

[CR3] Berman P, Karpinski M (1999) On some tighter inapproximability results (extended abstract). In: International Colloquium on automata, languages and programming (ICALP), Lecture notes in computer science, vol 1644, pp 200–209

[CR4] Budden D, Jones M (2017). Cautionary tales of inapproximability. J Comput Biol.

[CR5] Cavender JA (1978). Taxonomy with confidence. Math Biosci.

[CR6] Fakcharoenphol J, Kumpijit T, Putwattana A (2015) A faster algorithm for the tree containment problem for binary nearly stable phylogenetic networks. In: 2015 12th international joint conference on computer science and software engineering (JCSSE), IEEE, pp 337–342

[CR7] Farris JS (1973). A probability model for inferring evolutionary trees. Syst Biol.

[CR8] Fischer M, Van Iersel L, Kelk S, Scornavacca C (2015). On computing the maximum parsimony score of a phylogenetic network. SIAM J Discret Math.

[CR9] Fitch W (1971). Toward defining the course of evolution: minimum change for a specific tree topology. Syst Biol.

[CR10] Gambette P, Gunawan AD, Labarre A, Vialette S, Zhang L (2015) Locating a tree in a phylogenetic network in quadratic time. In: RECOMB, pp 96–107

[CR11] Griffiths RC, Marjoram P, Donelly P, Tavaré S (1997). An ancestral recombination graph. Progress in population genetics and human evolution.

[CR12] Gunawan AD, Lu B, Zhang L (2016). A program for verification of phylogenetic network models. Bioinformatics.

[CR13] Gusfield D (2014). ReCombinatorics: the algorithmics of ancestral recombination graphs and explicit phylogenetic networks.

[CR14] Hein J (1990). Reconstructing evolution of sequences subject to recombination using parsimony. Math Biosci.

[CR15] Huson DH, Rupp R, Scornavacca C (2010). Phylogenetic networks: concepts, algorithms and applications.

[CR16] Jin G, Nakhleh L, Snir S, Tuller T (2006). Maximum likelihood of phylogenetic networks. Bioinformatics.

[CR17] Jin G, Nakhleh L, Snir S, Tuller T (2007). Efficient parsimony-based methods for phylogenetic network reconstruction. Bioinformatics.

[CR18] Kanj IA, Nakhleh L, Than C, Xia G (2008). Seeing the trees and their branches in the network is hard. Theor Comput Sci.

[CR19] Kannan L, Wheeler WC (2012). Maximum parsimony on phylogenetic networks. Algorithms Mol Biol.

[CR20] Kelk S, Scornavacca C (2014). Constructing minimal phylogenetic networks from softwired clusters is fixed parameter tractable. Algorithmica.

[CR21] Morrison D (2011). Introduction to phylogenetic networks.

[CR22] Nakhleh L, Jin G, Zhao F, Mellor-Crummey J (2005) Reconstructing phylogenetic networks using maximum parsimony. In: Computational systems bioinformatics conference, 2005. Proceedings. 2005 IEEE, IEEE, pp 93–102 10.1109/csb.2005.47 16447967

[CR23] Nordborg M (2001) Coalescent theory. In: Balding, DJ , Bishop, M and Cannings, Christopher, Wiley, Hoboken

[CR24] Papadimitriou CH, Yannakakis M (1991). Optimization, approximation, and complexity classes. J Comput Syst Sci.

[CR25] Roch S (2006). A short proof that phylogenetic tree reconstruction by maximum likelihood is hard. IEEE/ACM Trans Comput Biol Bioinform.

[CR26] Tuffley C, Steel M (1997). Links between maximum likelihood and maximum parsimony under a simple model of site substitution. Bull Math Biol.

[CR27] Van Iersel L, Semple C, Steel M (2010). Locating a tree in a phylogenetic network. Inf Process Lett.

[CR28] van Iersel L, Jones M, Scornavacca C (2018) Improved maximum parsimony models for phylogenetic networks. Syst Biol 67(3):518–542 10.1093/sysbio/syx094 29272537

[CR29] Vuilleumier S, Bonhoeffer S (2015). Contribution of recombination to the evolutionary history of hiv. Curr Opin HIV AIDS.

[CR30] Wen D, Yu Y, Nakhleh L (2016). Bayesian inference of reticulate phylogenies under the multispecies network coalescent. PLoS Genet.

[CR31] Yu Y, Degnan J, Nakhleh L (2012). The probability of a gene tree topology within a phylogenetic network with applications to hybridization detection. PLoS Genet.

[CR32] Zhang C, Ogilvie HA, Drummond AJ, Stadler T (2017). Bayesian inference of species networks from multilocus sequence data. Mol Biol Evol.

[CR33] Zhaxybayeva O, Doolittle WF (2011). Lateral gene transfer. Curr Biol.

